# Evaluation of OPTOS wide‐field fundus image projections for radiotherapy planning of uveal melanoma

**DOI:** 10.1002/acm2.70009

**Published:** 2025-02-17

**Authors:** Jörg Wulff, Benjamin Koska, Michael Giese, Christian Bäumer, Ronald Richter, Andreas Foerster, Nikolaos E. Bechrakis, Beate Timmermann

**Affiliations:** ^1^ West German Proton Therapy Centre Essen (WPE) Essen Germany; ^2^ University Hospital Essen Essen Germany; ^3^ West German Cancer Centre (WTZ) Essen Germany; ^4^ CoCreation Lab Produktinnovationen (CCLP) University Duisburg‐Essen Essen Germany; ^5^ German Cancer Consortium (DKTK) Essen Germany; ^6^ Department of Physics TU Dortmund University Dortmund Germany; ^7^ Department of Ophthalmology University Hospital Essen Essen Germany; ^8^ Department of Particle Therapy University Hospital Essen Essen Germany

**Keywords:** 3D printing, Fundus imaging, proton therapy

## Abstract

**Purpose:**

To investigate the relationship between the stereographic and azimuthal equidistant projection (AEP) of the human retina for radiotherapy planning with OPTOS optomap wide‐field fundus images (Optos, UK). Further, the geometric accuracy of an OPTOS fundus image is quantified.

**Methods:**

The fundamental relationship between both projection modes was applied to transform images acquired with an OPTOS Silverstone camera to the azimuthal equidistant projection using MATLAB. Fundus images of four patients were used to quantitatively demonstrate the impact for neglecting the proper projection. For that purpose, a delineated contour for each patient was analyzed if created in a treatment planning system, which assumes AEP, and compared with an OPTOS image. Furthermore, an eye model with a novel 3D printed retina pattern was used to quantify the geometric accuracy for an OPTOS optomap image.

**Results:**

The difference between both projections was found substantial, leading to a change in delineated contours of more than 5 mm in the investigated cases and a change of delineated area of more than 40%. The geometric accuracy of OPTOS images of a customized eye model was found to be 0.2 mm on average, increasing to at most ∼0.5 mm at eye angles of 81°.

**Conclusion:**

The fundamental difference in the representation of the eye fundus needs to be accounted for in radiotherapy planning of uveal melanoma. The basic underlying relationship for transformation is known, but more research is required to quantify other aberrations. The novel use of 3D printed retina patterns with known dimensions is providing a flexible approach for further investigations.

## 1 INTRODUCTION

Fundoscopy imaging plays a fundamental role in the diagnosis of ocular disease.^1^ Fundus images are also used in radiotherapy planning.^2^, ^3^, ^4^, ^5^ Currently, there are only a few treatment planning systems (TPSs) allowing direct incorporation of fundus images in the planning/delineation process, for example, EYEPLAN,^6^ OCTOPUS,^7^ and RayStation (RaySearch Laboratories, Sweden) for external beam therapy and the Plaque Simulator (Eye Physics LLC, USA) for brachytherapy.^8^ These treatment planning systems all display the planned dose distribution in a fundus view, which can also serve to overlay post‐treatment fundus images.

One of the most common manufacturers of wide‐field cameras is OPTOS (Optos, UK) with an installed base of over 25 000 devices worldwide according to the manufacturer. The images of the OPTOS scanning laser‐ophthalmoscope (SLO) cameras allow for a field‐of‐view of up to 200° in a single image.^9^ Similar to EYEPLAN, the RayOcular TPS in RayStation version 2024 allows the overlay of a fundoscopy image to a fundus view, which projects the 3D eye model into a flat 2D image representation. The so‐called “polar mode” of RayOcular and EYEPLAN is an *azimuthal equidistant projection* (AEP), while the OPTOS images are represented in a *stereographic projection* with differences increasing with distance from the posterior pole.^10^ The fundamental difference in the projection modes may jeopardize any accurate target delineation on OPTOS wide‐field fundus images within a TPS when not corrected for. It needs to be noted that the conversion between both projection methods was introduced in the brachytherapy Plaque Simulator already in its version 6.7.9 from January 2021. A similar feature is however not available in the TPS for proton therapy yet.

The overall accuracy of the OPTOS wide‐field image called *optomap* is generally difficult to quantify, as no human ground truth exists. Sagong et al. compared optomap images with known dimensions of an intraocular prosthesis.^11^ They demonstrated that areal measurements in the images agree to within a few percent from specifications of the prosthesis, with approximately 3 mm side‐length. On the other hand, Muttuvelu et al. observed inconsistent distortions in OPTOS images in repeated scans.^12^

The purpose of this work is two‐fold. The fundamental relationship between the stereographic and AEP is investigated and a transformation approach between both is evaluated. The impact of the differences is demonstrated for a set of clinical cases. Further, the geometric representation accuracy of optomap wide‐field images is tested. For that purpose, a mechanical eye model is equipped with a 3D printed retina pattern of known dimensions.

## 2 MAP PROJECTIONS

Projecting a spherical, curved surface to a flat image is historically known from cartography. Different projections exist,^13^ which preserve some property of the surface, for example, shape, area, etc., but any projection inevitably entails some distortions.

Azimuthal projections may be considered a natural choice for displaying the retina. The center of the projection corresponds to the posterior pole of the eye. The scale from the center is radially symmetrical, that is, points on, for example, the eye's equator can be found on a circle in the projection. Two types of *azimuthal* projections are of interest for this study: the *stereographic* and the *equidistant*. Both projections are conformal, that is, angles and, thus, shapes are preserved in the projection. In case of the AEP (= azimuthal equidistant projection), a distance from the posterior pole is directly proportional to the eye angle (i.e., latitude relative to the posterior pole), while for the stereographic projection, the distance is proportional to a *tan*‐function of the angle. Structures in a stereographic projection image are thus enlarged toward the equator compared to an AEP.

**FIGURE 1:**
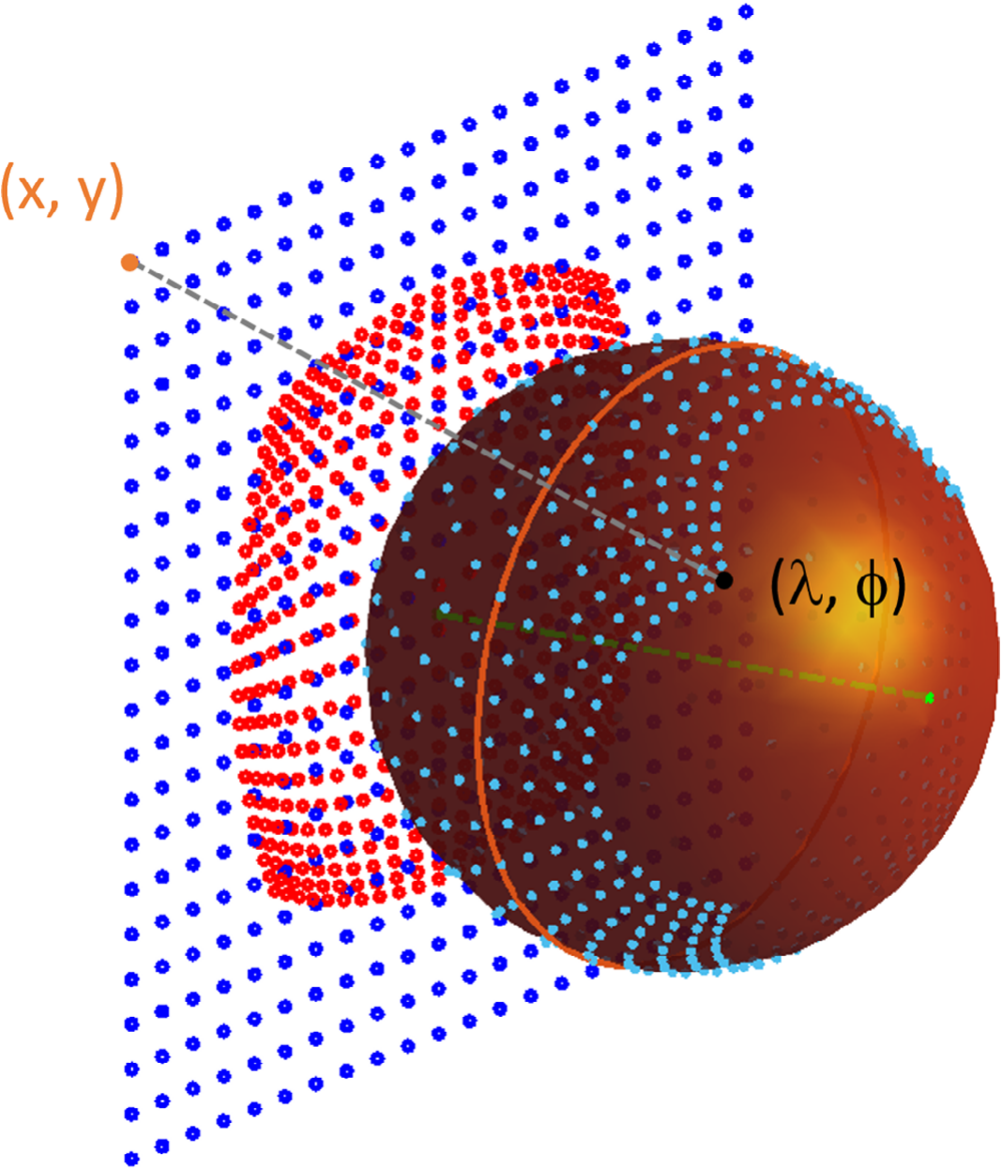
Example of a stereographic projection (blue dots) and AEP (red dots) for a spherical surface. The equidistant points on the projection plane with coordinates (x,y) correspond to points on the spherical surface at (λ, ϕ). The orange line represents the equator. The green line indicates the connection between both poles and passes the origin at (λ_0_, ϕ_0_) to the center pixel.

For both projections and its inverse, exact formulations exist for a sphere.^13^ The OPTOS wide‐field camera follows the DICOM wide‐field standard.^14^ The fundus image, which is initially defined by the camera system of lenses, mirrors, and other optical elements is eventually represented in a stereographic projection. This projection combines an optical model of the camera and the standardized model of the human eye by Navarro with axial length of 24 mm.^15^, ^16^, ^17^ The DICOM standard also describes the inverse projection from a 2D image in pixel space to the 3D coordinates on a (unit) sphere. Figure 1 shows exemplarily how points in a flat, uniformly spaced image are mapped to a spherical surface following the descriptions in the DICOM standard. The points on the surface of the sphere can then be transformed to any other map projection, for example, the AEP indicated as red dots in Figure 1.

## 3 MATERIALS AND METHODS

### 3.1 Image transformations

A conversion between the stereographic projection in an OPTOS Silverstone wide‐field optomap image and the AEP projection was implemented in MATLAB v2023b (The Mathworks, USA).

First, the transformation between the pixel‐coordinates in the OPTOS image to the spherical angles λ (azimuth/ longitude) and ϕ (elevation/ latitude) followed the equations given in the DICOM wide‐field standard (see section C.8.17.11.1.1 therein). The Center Pixel View Angle property of the optomap image was taken with 0.08596515° as defined in the DICOM image (0022,1528) of the OPTOS Silverstone camera with resolution for the 4000 times 4000 pixels. From the spherical coordinates, a forward AEP followed the formulations in Ref.[13]

The azimuthal (and stereographic) projections depend on the origin and center of projection defined by λ_0_ and ϕ_0_. In its simplest form, the coordinate matches the pole, that is, opposite to the corneal apex. A re‐projection can also be performed for a different center and thus angle of the central ray through the center of the sphere (see Figure 1). The stereographic projection itself may need to be re‐projected to another center. This was the case for images taken with the eye model (see Section 3.2) where the center of the image was not coinciding with the center of the model due to non‐ideal alignment angle during acquisition. In the AEP, a different center of projection may be required as the posterior pole is not visible in the image, but has a defined angle relative to the treatment planning eye model. To allow a proper overlay in the TPS retinal/fundus view, the re‐projection to the azimuthal equidistant space needs to match. Therefore, the AEP and stereographic re‐projection functions included the definition of the center in spherical angles λ_0_ and ϕ_0_.

Finally, a 1‐to‐1 correspondence between pixel locations in the stereographic and equidistant projection can be established, which was the basis of a 2D transformation of pixels in the original image using the imwarp function in MATLAB. The function was used with a piece‐wise linear 2D image transformation with the AEP of every 200 pixels as control‐points defining a displacement field.

### 3.2 Validation eye model

**FIGURE 2:**
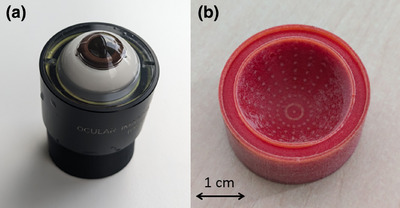
Image of the OEMI‐7 eye model (a) and the 3D printed retina cup (b).

As no ground truth in vivo can be established, the commercially‐available Ocular Instruments (Ocular Instruments, USA) OEMI‐7 mechanical eye model (Figure 2a) was used to investigate the imaging with the OPTOS Silverstone camera. This water‐filled eye model mimics an emmetropic human eye with a total power of 60 diopters and consists of a cornea, pupil, polymethyl‐methacrylate crystalline lens, aqueous and vitreous cavities, as well as a retinal surface with a curvature radius of 11.9 mm.^18^ The modular design of the model allows to exchange the retina, designed as a hemi‐sphere “cup.”

A 3D printed version of the retina cup was reproduced from the retina cup of the delivered version in terms of shape and size. Small half‐spheres with a radius of 0.2 mm were embedded in the surface and printed in yellow, while the rest of the model was printed in red. The marks were distributed in a 9 × 20 grid, every 9° from the posterior pole in latitude and 20° in azimuthal dimension (Figure 2b). Thus, the model allowed imaging of the retina for straight orientation of the model up to the equator. The 3D model was desinged in Rhinoceros 3D CAD software v 8 (Robert McNeel & Associates, USA) and then converted with the Stratasys software GrabCAD, where the printing parameters and colors were defined.

3D printing was performed in PolyJet technique (resin‐based UV‐curing technology) on a J55 Stratasys printer (Stratasys, USA). The cup was printed with Vero Color resins from Stratasys including a water soluble support material. The support was removed afterwards in a water bath. No further post‐processing was applied.

### 3.3 Image transformations in clinical examples

As elucidated in the introduction, the TPS may display the image in an AEP. If a tumor base is delineated in this view with a stereographic projection optomap image, the size of the resulting structure will be overestimated. To demonstrate the impact of the two projection modes, four patients with uveal melanoma were selected. The melanoma base of each case was marked as a polygon in MATLAB on the OPTOS image. The points of the polygon were mapped to a spherical surface (2.4 cm diameter) with an inverse azimuthal projection and the inverse stereographic projection. The difference in largest diameter of the contour and the area were calculated in MATLAB. For visualization purposes, the points on the sphere from inverse azimuthal projection were re‐projected to the stereographic image and overlaid.

### 3.4 Accuracy quantification in OPTOS wide‐field images of the eye model

The deviation between the nominal positions from the 3D printed retina to the displayed location in the 2D stereographic image was quantified. The pixel locations of the retina pattern points in the DICOM image were manually identified. Each pixel location was mapped to the surface of a sphere and compared to the nominal coordinates in terms of angles λ and ϕ. Initially, the center of the DICOM image was not coinciding with the center of the pattern. This was interpreted as a small remaining angle during acquisition of the image, and thus, the spherical angles of the pattern were corrected to match the center in the image. Finally, the remaining shortest distance (geodesic) on a sphere with 2.4 cm diameter between the nominal and identified point was calculated using the Vincenty formula.^14^

## 4 RESULTS

### 4.1 Image transformations

**FIGURE 3:**
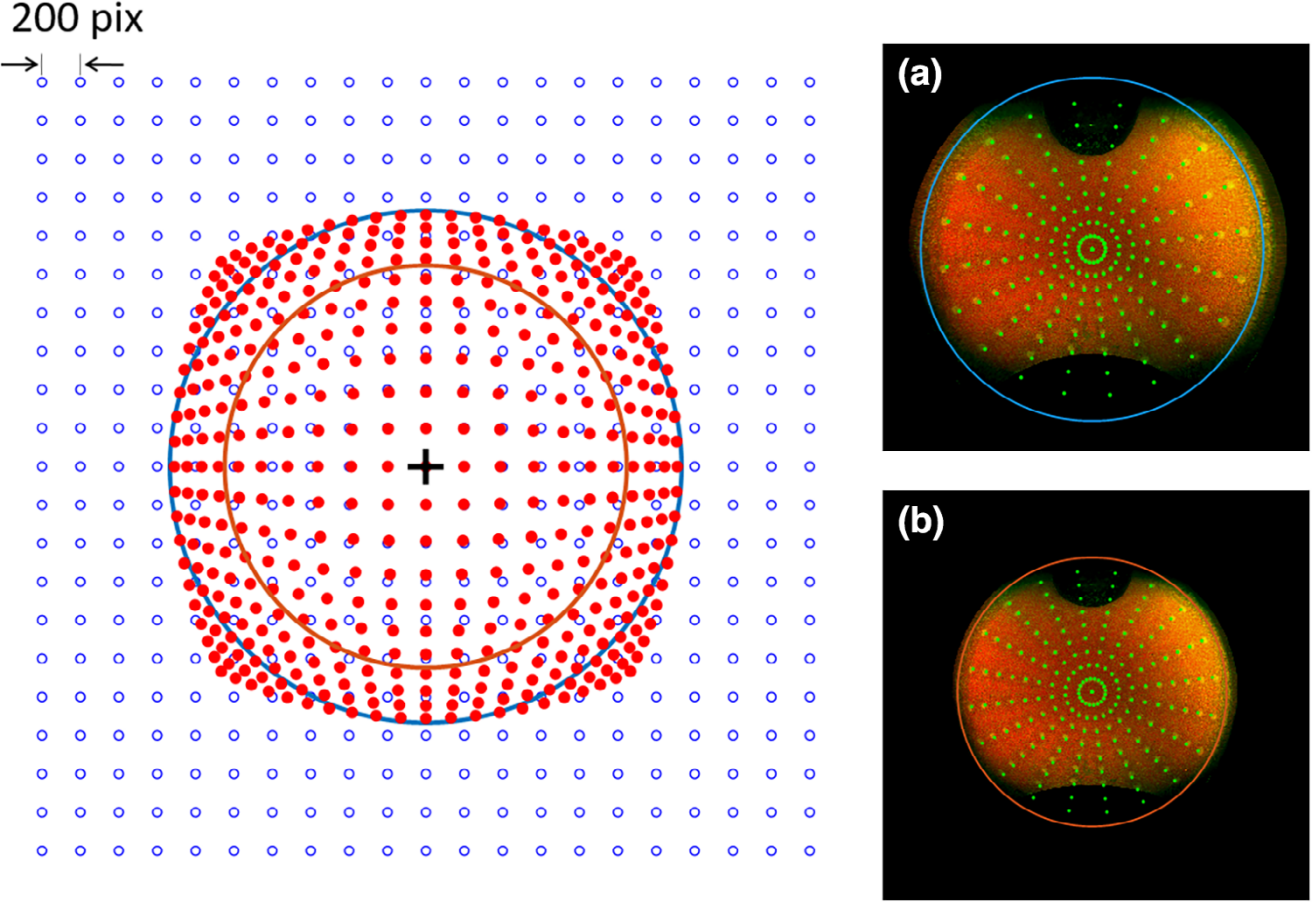
Sample points in the stereographic projection (blue open dots) mapped to AEP of the same points (red filled). The solid lines corresponds to the equator of the sphere in both projection modes. On the right hand side, the resulting transformation from stereographic representation (a) to azimuthal equidistant (b) is shown for the eye model with retina pattern. In both cases, a 4k x 4k pixel image is shown. The circles represent the projection of equator points of an underlying sphere model to the respective 2D projection. The green dots indicate the projection of the nominal pattern points to the respective representation.

Figure 3 illustrates the difference between the stereographic projection and the AEP. Due to the *tan*‐function proportionality in the stereographic projection, the deviation increases with angle from the posterior pole, that is, toward the edges of the image. The transformation matrix was based on these control points in both projections and applied to OPTOS images. Figure 3a shows a sample optomap image of the eye model acquired with the OPTOS Silverstone camera together with the nominal position of the retina pattern. Figure 3b is the corresponding image after transformation to AEP. Again, the nominal positions of the retina pattern as well as the equator in the AEP are overlaid. There is a limited field of view at the lower part of the image due to the OPTOS SLO design. The upper shadowing of the image was caused by a small air‐bubble in the anterior chamber of the eye model.

**FIGURE 4:**
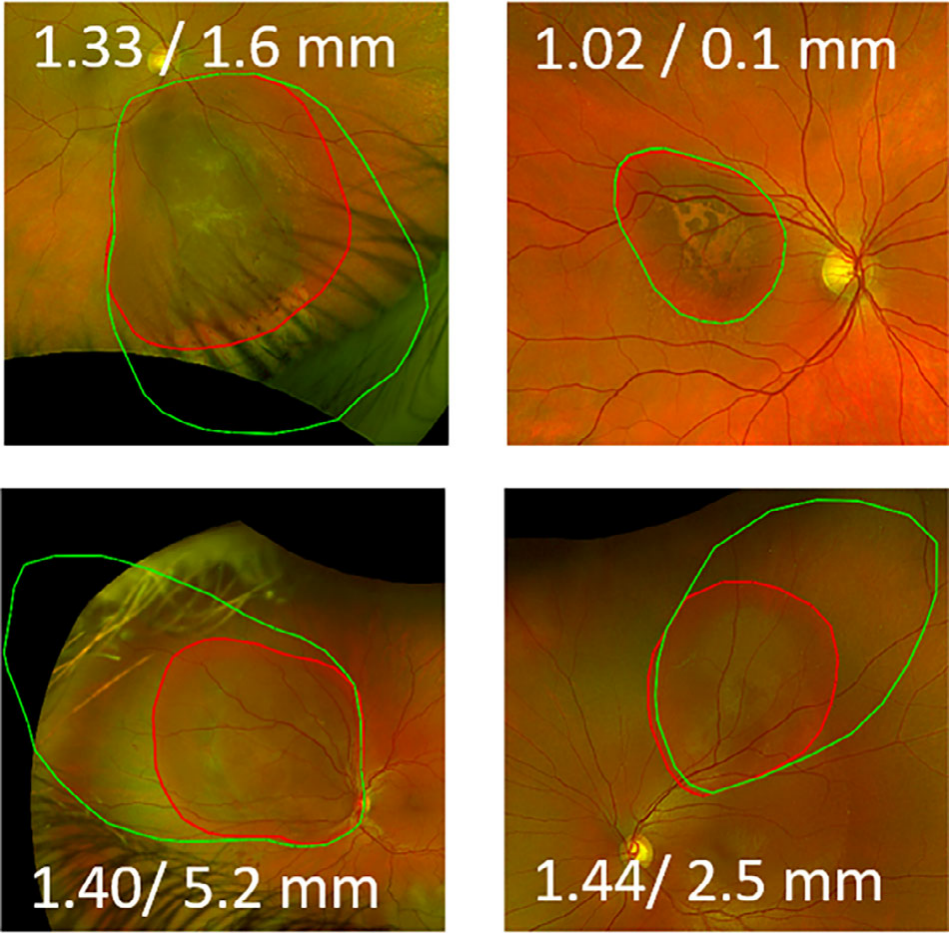
Clinical examples of uveal melanoma delineation (red). The green line corresponds to a re‐projection from azimuthal equidistant to stereographic projection. The numbers in each image indicate the relative change of delineated area and the absolute difference of largest diameter on a sphere with 2.4 cm diameter.

Figure 4 shows the selected four clinical examples with a uveal melanoma. The red line indicates the base of the tumor as delineated from this image. If this delineation is done in a model based TPS that displays the retina in an AEP overlaid with the optomap image, the resulting melanoma dimensions are overestimated. This is illustrated by re‐projecting the points of the polygon from azimuthal equidistant to stereographic projection and overlaying to the same image (green line in Figure 4). Depending on the extent of the melanoma toward the equator, the area can be overestimated by up to a factor of 1.44. The largest diameter of the delineation differs by up to 5.2 mm for one of the cases.

### 4.2 Accuracy quantification in OPTOS wide‐field images of the validation eye model

**FIGURE 5:**
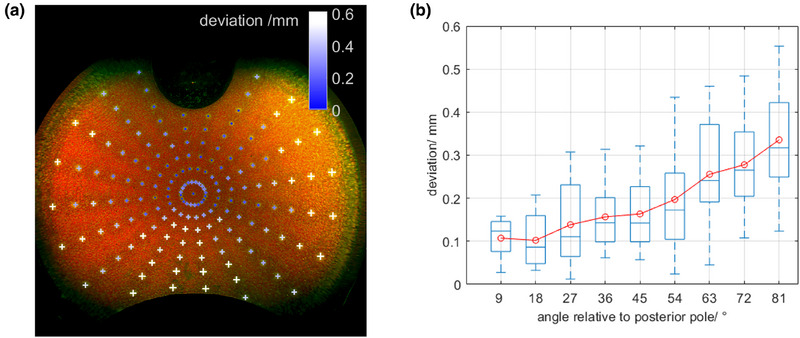
Stereographic projection of the eye model image taken with the OPTOS Silverstone camera. (a) The positions of the 3D printed retina pattern is overlaid as colored crosses with color and relative size corresponding to geodesic to nominal positions. (b) Boxplots of all deviations (geodesic) between nominal positions of pattern and measured positions of retina pattern as a function of eye angle relative to the posterior pole. The red circles correspond the average offset.

The difference between the displayed points of the retina pattern in the optomap image and their expected locations was 0.2 mm on average with an increasing trend towards the periphery, that is, toward the equator. Figure 5a shows the OPTOS image with the pattern of points on the eye model retina. Figure 5b depicts remaining deviations as a function of the eye angle relative to the posterior pole.

Some images were taken with a tilt of the eye model during acquisition, that is, the light of the camera entering at a significant angle. In that case, the overlay of the retina pattern resulted in discrepancies of several millimeters.

## 5 DISCUSSION

If not corrected for, the deviation between the AEP in the radiotherapy treatment planning system and the stereographic projection in the OPTOS optomap image may be significant. If no other source of information is used in the delineation process, this could lead to an unnecessary large target volume. However, in proton therapy, a multimodal imaging approach is followed and tantalum fiducials are sutured to the outer sclera prior to treatment planning, guiding the delineation process.^19^ Further, the increased dimensions of the target base occur toward the equator. A single anterior proton beam configuration will have minor clinical consequences, as the proximal dose is typically high. If external radiotherapy with lateral beams is applied, such as in stereotactic photon treatments, over‐treating the anterior segment may result in unnecessary high toxicities, for example, to the lens or ciliary body. If brachytherapy planning is based on fundus images and the projection differences were not corrected for, mismatch would be obvious during the actual placement of the plaque. As mentioned, at least the Plaque Simulator software includes the conversion of optomap images to AEP.

Recently, Fleury et al. proposed a Mercator projection for displaying the dose distribution in Cyberknife treatments of uveal melanoma^20^ and one could also re‐project the OPTOS optomap image to match this type of projection. The distortion effects may also be relevant for treatment assessment, that is, overlaying post‐treatment fundus images with the planned dose distribution.

Based on the eye model image analysis, the deviation between the expected nominal positions in the retina pattern and the locations in the OPTOS optomap image was 0.2 mm on average, increasing toward the equator. The remaining differences can be attributed to limitations in the eye model itself, that is, in the design of the anterior part/lens, the non‐spherical shape and total length not perfectly matching the underlying eye model in the OPTOS optomap processing. There may also occur some aberrations in the camera optics itself. A variation between the properties of the human eye and the underlying optomap model to some degree is expected in a clinical situation, so the reported agreement may be considered a lower uncertainty estimate for delineation in a TPS. It needs to be noted that the reported agreement of the retina pattern was the case for a straight oriented eye model. For a tilted eye, the deviations were much larger and point toward limitations of the imaging and/or eye model, but also to possible variability in clinical examinations. This may also be the case for the observed discrepancies in Muttuvelu et al.^12^ Generally, a minimal tilt during acquisition appears advisable.

Generally, there are further challenges in accurately reconstructing the 3D eye and tumor from a fundus image. Other higher order aberrations may occur, for example, due to the individual lens refraction or non‐spherical eye anatomy.^21^ It is not clear how, for example, an artificial lens or deformations of the retina due to retinal detachment impact the projection. More research is required, but with better knowledge of the individual eye, one can envision a re‐projection through the exact anatomy using sophisticated mathematical eye models,^22^, ^23^ which could reduce the associated uncertainties.

The method of investigating the camera projection with the 3D printed pattern could also be applied to other fundoscopy systems. However, while the optomap stereographic projection is defined in the DICOM wide‐field standard,^14^ other systems may include parameters in their projection, which are not directly available to a user. This does not impact measurements performed in the cameras‘ software, but the re‐projection is less straightforward.^10^

## 6 CONCLUSION

The fundamental difference between the azimuthal and stereographic projection should be accounted for if OPTOS images are used in the treatment planning system with a “polar mode” fundus projection. The reported projection effects can be investigated with an example function provided in the data sharing repository. A functionality for a direct conversion within the TPS following the DICOM standard definitions of wide‐field images as described in study is desirable. Ideally, all vendors of fundoscopy cameras would then provide the images in the wide‐field format.

The OPTOS optomap image was demonstrated to accurately represent the geometry of the customized eye model within 0.2 mm on average. Given the flexibility of the eye model in combination with the 3D printing approach, more eye models can be designed to include anatomical variations to further investigate the fundus imaging of human eyes.

## AUTHOR CONTRIBUTIONS

**Jörg Wulff**: Conceptualization; methodology; investigation; analysis; writing—original draft; visualization; project administration. **Benjamin Koska**: Conceptualization; methodology; investigation; analysis; writing—review & editing. **Michael Giese**: Methodology; technical support; writing—review & editing. **Christian Bäumer**: Conceptualization; methodology; investigation; analysis; writing—review & editing. **Ronald Richter**: Conceptualization; investigation; analysis; writing—review & editing. **Andreas Foerster**: Conceptualization; investigation; analysis; writing—review & editing. **Nikolaos E. Bechrakis**: Conceptualization; investigation; writing—review & editing. **Beate Timmermann**: Conceptualization; investigation; writing—review & editing

## CONFLICT OF INTEREST STATEMENT

The authors declare no conflicts of interest

## ETHICS STATEMENT

Images shown belong to patients who were enrolled in a prospective registry study (“ProReg,” German Clinical Trial Register: DRKS00004384), which is covered by an ethics approval. Patients had provided written informed consent.
